# Who was the real sabertooth predator: *Thylacosmilus* or *Thylacoleo*?

**DOI:** 10.1002/ar.25444

**Published:** 2024-04-10

**Authors:** Christine M. Janis

**Affiliations:** ^1^ Palaeobiology Group, School of Earth Sciences University of Bristol Bristol UK; ^2^ Department of Ecology, Evolution and Organismal Biology Brown University Providence Rhode Island USA

**Keywords:** ecomorphology, sabertooths, *Thylacoleo*, *Thylacosmilus*

## Abstract

Sabertoothed mammalian predators, all now extinct, were almost exclusively feloid carnivorans (Eutheria, Placentalia): here a couple of extinct metatherian predators are considered in comparison with the placental sabertooths. *Thylacosmilus* (the “marsupial sabertooth”) and *Thylacoleo* (the “marsupial lion”) were both relatively large (puma‐sized) carnivores of the Plio‐Pleistocene in the Southern Hemisphere (Argentina and Australia, respectively). Both carnivores have captured the public imagination, especially as predators that were somehow analogous to northern placental forms. But a more detailed consideration of their morphology shows that neither can be simply analogized with its supposed placental counterpart. While *Thylacosmilus* did indeed have saber‐like canines, many aspects of its anatomy show that it could not have killed prey in the manner proposed for the sabertoothed felids such as *Smilodon*. Rather than being an active predator, it may have been a specialized scavenger, using the hypertrophied canines to open carcasses, and perhaps deployed a large tongue to extract the innards. *Thylacoleo* lacked canines, and its supposedly “caniniform” incisors could not have acted like a felid's canines. Nevertheless, while its mode of dispatching its prey remains a subject for debate, it was clearly a powerful predator, likely to be capable of bringing down prey bigger than itself while hunting alone. In that regard, it may have filled the ecomorphological role proposed for placental sabertooths, and so despite the lack of canines can be nominated as the true “marsupial sabertooth” out of the two extinct taxa.

## 1 INTRODUCTION

Large carnivores have always had a strong public appeal, and large cats especially so, from the “king of the jungle” onwards. Sabertoothed carnivores, no longer with us, appear to have been a type of super‐predator with hypertrophied canine teeth, and so feature heavily in people's imagination as heroic “sabertoothed tigers” (why tigers rather than lions, one might wonder: perhaps lions, with the mane in the males, are perceived as more cuddly).

What does it mean to be a “sabertooth” in terms of predatory behavior? Were all extinct carnivores with hypertrophied canines the same type of predator? And is it possible that some extinct carnivores were similar to sabertooths in their probable predatory role, despite lacking the dental equipment? Here, I consider the sabertooth ecomorphological type in relation to the extinct metatherian carnivores *Thylacosmilus* (the “marsupial sabertooth”) and *Thylacoleo* (the “marsupial lion”) and discuss their probable predatory mode in the relation to the one proposed for placental feloid sabertooths. This paper is largely a summary of the previous work of the author and colleagues (Figueirido et al., 2016; Janis et al., 2020), along with some comments on more recent papers.

### 1.1 Introducing cat‐like metatherians

**FIGURE 1:**
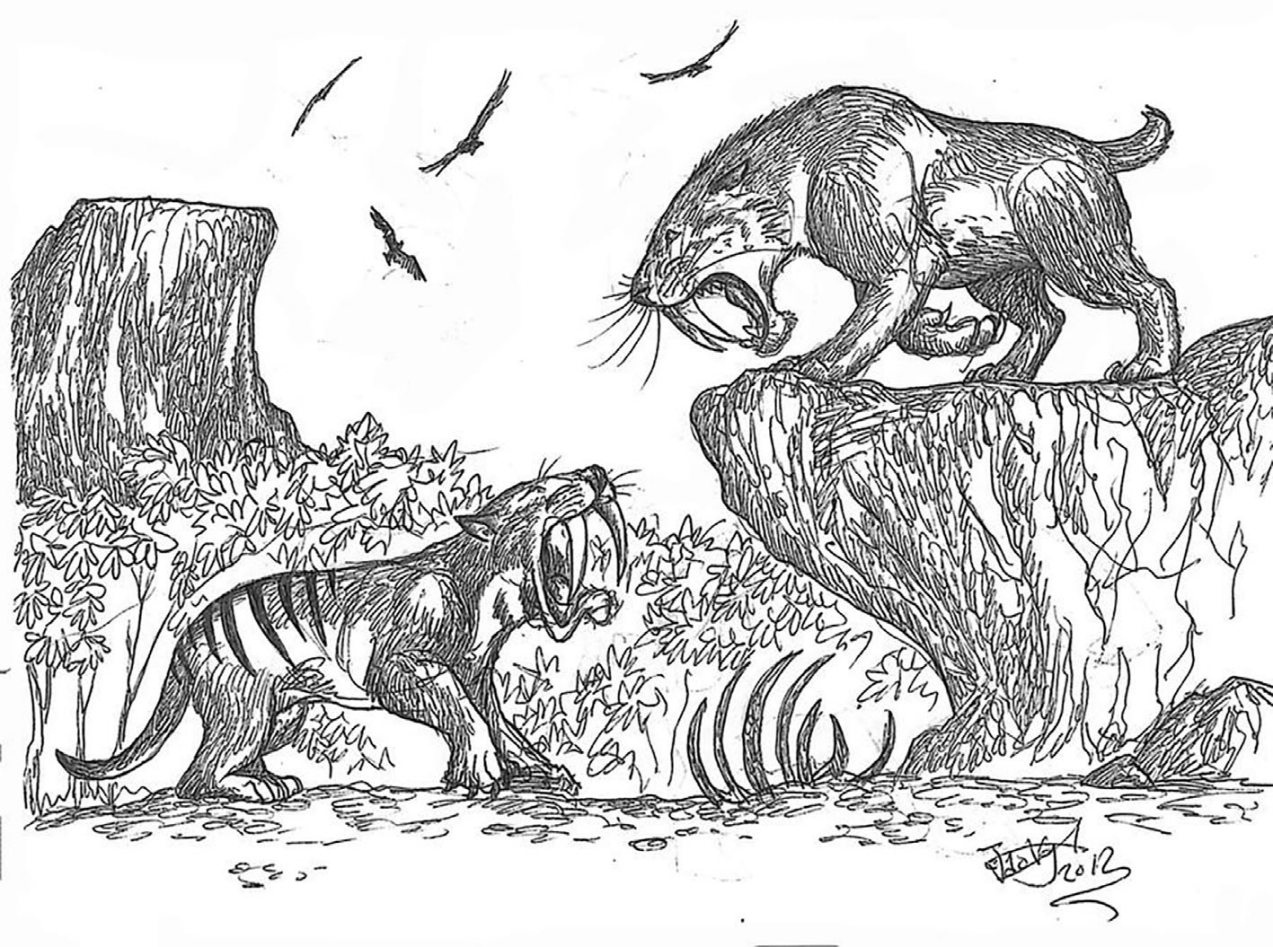
“Rise of *Smilodon*” by hodarinundu, depicted here threatening a *Thylacosmilus*. Reproduced by permission of the artist.

The known sabertoothed mammals are almost exclusively feloids (Carnivora) of some sort, evolving convergently at least three times in the Felidae (Machairodontinae: Pleistocene) Nimravidae (late Eocene to Miocene) and Barbourofelidae (Miocene, sometimes included within the Nimravidae, see Barrett, 2021). A couple of small forms (with relatively small canines) are also known from the Eocene of North America among the Oxyaenidae (Creodonta, possibly related to the Carnivora). Meanwhile, a popular candidate for a marsupial version is the South American (Pliocene of Argentina) *Thylacosmilus atrox* (Sparassodonta, Borhyaenoidea, Thylacosmilidae—strictly a metatherian rather than a marsupial as sparassodonts fall outside of crown‐group Marsupialia). This animal is often described as having lost out to the “real” sabertooth, *Smilodon populator* (Felidae, Machairodontinae) when the latter animal arrived from the northern American continent in the Pleistocene—despite the fact that *Smilodon* arrived in South America at least a million years after *Thylacosmilus* had gone extinct (see Prevosti et al., 2013) (see Figure 1).

Another well‐known extinct cat‐like metatherian (this time also a marsupial), from the Pleistocene of Australia, is *Thylacoleo carnifex* (Diprotodontia, Thylacoleonidae), the so‐called “marsupial lion.” This animal has not captured the popular imagination quite as much as *Thylacosmilus*, perhaps because it lacks canines (apart from tiny nubbins), and so has never been considered as an analog of sabertoothed felids (although it has been considered to be a sabertooth‐like “super‐predator”; Wroe et al., 2008). Despite the lack of a canine killing arsenal, *Thylacoleo* had prominent incisors (said to be “caniniform,” but see later discussion), and a prominent claw on its pollux (thumb), protected by an extensive sheath, with an apparently swivel‐like joint between the thumb and the wrist (Wells & Nichol, 1977), which may have been an equally impressive predatory tool as a large canine (see Figueirido et al., 2016).

**FIGURE 2:**
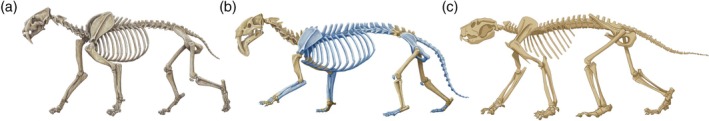
Skeletons of three cat‐like carnivores. (a) *Smilodon fatalis*, (b) *Thylacosmilus atrox*, (c) *Thylacoleo carnifex*; (a) and (b) by Mauricio Anton, reproduced here by permission of the artist. (c) Modified from Wells and Camens (2018), fig. 11B, redrawn by Science Graphic Design (sciencegraphicdesign.com). Original drawing by Peter Murray, reproduced here with permission from Rod Wells. Blue shading indicates unknown bones.

Both of these cat‐like metatherians were around the size of a large puma (~100 kg: *Thylacoleo*, Wroe et al., 1999; *Thylacosmilus*, Ercoli & Prevosti, 2011; Wroe et al., 2013, although see a much lower estimate of ~40 kg in Suarez et al., 2023). Thus they were around half the size of the iconic “sabertoothed tiger” *Smilodon fatalis* (~250 kg, Christiansen & Harris, 2005). Both metatherians have been compared to cats because of their overall appearance: short‐faced and powerfully built (although note that the skull of *Thylacosmilus* has some profound differences from any placental carnivore, Goswami et al., 2010). Both metatherians share the feature of a postorbital bar, an unusual anatomy for a carnivorous mammal, otherwise seen only in the placental feloid carnivoran *Barbourofelis*. The cat‐like short face would result in a greater mechanical advantage at the anterior end for the deployment of incisors and canines (Maynard Smith & Savage, 1959; Mitchell et al., 2023), and this likely explains the reason for a short face in these metatherian predators. Both metatherians differ from felids in having a back that is stiff rather than flexible, and a plantigrade (rather than digitigrade) foot posture (Argot, 2004; Wells & Camens, 2018), indicating that they were less cursorial than even a specialized (dirk‐toothed) sabertoothed felid (see Figure 2).

## 2 WHAT DOES IT MEAN TO BE A SABERTOOTH PREDATOR?

The sabertooth ecomorph is commonly supposed to represent a super‐ambush predator capable of bringing down prey as large or larger than itself (Andersson et al., 2011). Sabertoothed predators have been divided into two different ecomorphological types: dirk‐toothed forms, with longer canines and a less cursorially built postcranial skeleton (e.g., *Smilodon*), and scimitar‐toothed forms, with shorter canines and a postcranial skeleton more like extant large felids (e.g., *Homotherium*) (Martin, 1980). Felids and nimravids contained both ecomorphs; barbourofelids were dirk‐toothed, as was *Thylacosmilus*. Especially among dirk‐toothed sabertooth felids (*Smilodon* being the classic example), these saber‐like canines are assumed to be involved in the killing of large prey and the robust forelimbs to be adapted for power rather than for speed indicative of good grappling abilities to subdue the prey (Meachen‐Samuels, 2012).

Extant felids kill their prey in a couple of different ways. For killing small prey, a bite to the nape of the neck breaks the spinal cord or the skull (Leyhausen, 1979); smaller prey items have more fragile bones and so the felid is less likely to suffer canine damage (Seidensticker & McDougal, 1993). For killing larger prey, extant felids apply a suffocating bite to the muzzle or the throat (Schaller, 1972). Both felid predation modes would have been liable to result in canine damage in sabertooths, especially in the case of the very long canines of dirk‐toothed forms, due to contact with bone in the first instance or torsion induced by struggling prey in the second instance (Salesa et al., 2005; Van Valkenburgh & Ruff, 1987).

**FIGURE 3:**
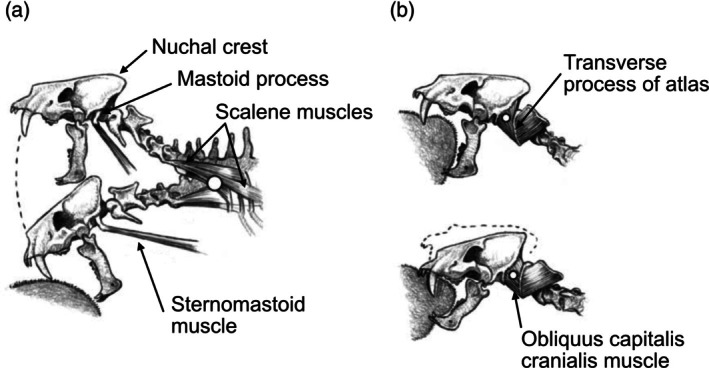
Proposed modes of killing by sabertoothed predators (shown on the felid *Homotherium latidens*). (a) Stabbing (= head strike). (b) Canine shear bite. The white circles show the point of the rotation of the head. Modified (muscle names added by Science Graphic Design [sciencegraphicdesign.com]) from Antón et al. (2004), fig. 1, with permission from Oxford University Press and Mauricio Antón.

Sabertooths are thus proposed to have used a different type of predatory bite, one which would employ the long canines to good advantage but which would minimize the risk of canine breakage. The proposed mode of killing was to initially pierce the soft tissues of the throat with a stabbing action or head strike: the head would first be elevated and then depressed, plunging the canines into the prey; then further depression of the head would drag the canines through the tissues, creating a canine shear bite along with closure of the lower jaw (see Antón et al., 2004 and Figure 3). This throat bite would sever the blood vessels and windpipe, resulting in rapid blood loss and suffocation. The rapidity of the killing bite (in contrast with the more prolonged suffocation bite of felids), along with the immobilization of the prey with powerful forelimbs, would aid in reducing the probability of canine damage (Salesa et al., 2005).

**FIGURE 4:**
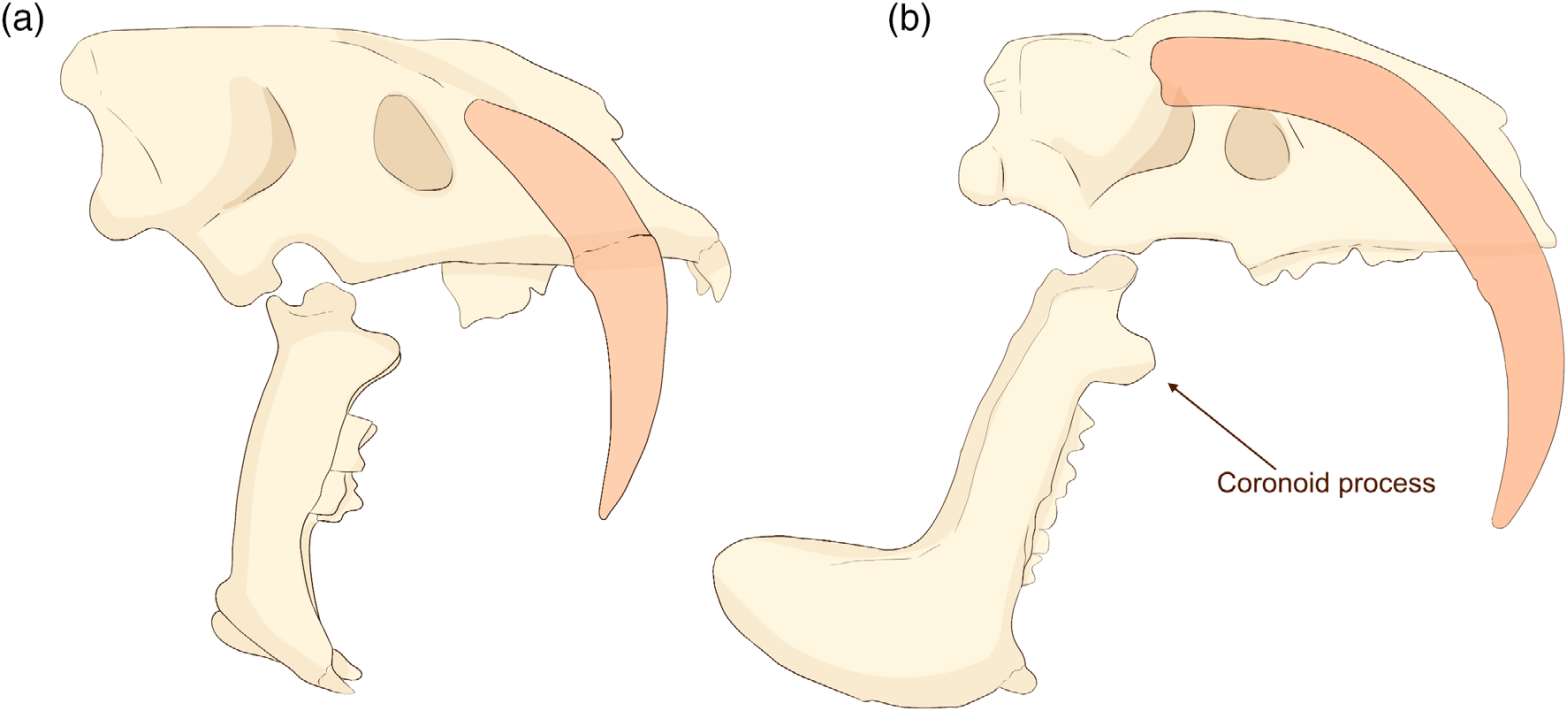
Comparison of gape in (a) *Smilodon fatalis* and (b) *Thylacosmilus atrox*. Modified from Wroe et al. (2013), fig. 1. Figure redrawn by Science Graphic Design (sciencegraphicdesign.com), with permission from Steven Wroe.

These actions would be reflected in the craniocervical anatomy, as indeed seen in sabertooth felids. The head strike is reflected in the following anatomical features (Figure 3a): a tall nuchal crest, acting as a lever arm for muscles to elevate the head; a large mastoid process, acting as a lever arm (and a greater area of origin) for the sternomastoid muscle, which is also aided by the action of the scalene muscles originating from the base of the neck. The canine shear bite, where the head alone is depressed, is also reflected in a large mastoid process, for the insertion of the obliquus capitalis cranialis muscles, and a large transverse process of the atlas, for the insertion of that muscle (Figure 3b) (Antón et al., 2004). In addition, sabertooths have adaptations for a wide gape, including a greatly reduced coronoid process, to allow for the lower jaw to be fully depressed during the head strike (see Figure 4) (Emerson & Radinsky, 1980).

## 3 WAS *THYLACOSMILUS* REALLY A SABERTOOTH ECOMORPH?

*Thylacosmilus* certainly had extraordinarily large canines, all the more remarkable for being ever‐growing, and a wider gape than sabertooth felids (with a correspondingly small coronoid process). Matching the enlarged upper canines, *Thylacosmilus* had large symphysial flanges that resembled those seen in most dirk‐toothed sabertooth felids (but absent in *Smilodon*) (see Figure 4). *Thylacosmilus* certainly looks like a sabertoothed felid, especially at first glance and in lateral view. But have we simply been blinded by the tooth?

### 3.1 The general shape of the skull

The overall shape of the skull of *Thylacosmilus* is certainly like that of other sabertooths, especially in the rostral and occipital/nuchal regions as shown by Melchionna et al. (2021). These authors note convergence in the shapes of sabertooth skulls, including and attribute this to similar prey‐catching behavior. However, many of these similarities could simply reflect adaptations for a wide gape and a powerful canine bite, which may not necessarily have been used to dispatch live prey. A short rostrum would confer a mechanical advantage at the canines and a robust muzzle would resist forces applied to the canines, while the need for a wide gape would result in morphologies such as the realignment of the jaw muscles and the lowering of the occipital condyles. The occipital area of *Thylacosmilus* is actually rather different from machairodont felids and *Barbourofelis*, as described below, and not indicative of the use of a headstrike action.

**FIGURE 5:**
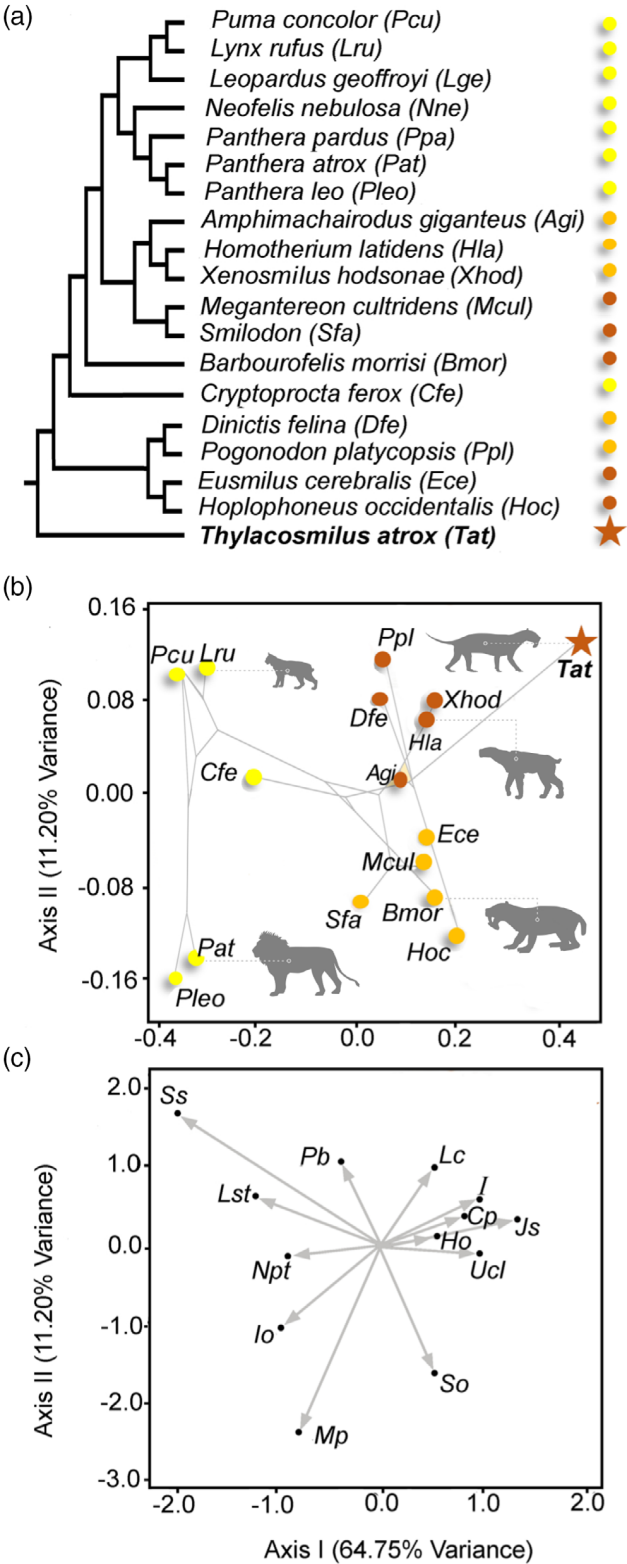
Canonical correspondence analysis of discrete morphological traits in felids and felid‐like carnivores. (a) Phylogeny employed. Yellow, conical‐toothed carnivores (extant felids and eupleurids); orange, scimitar‐toothed sabertooths (felids and nimravids); red, dirk‐toothed sabertooths (felids, nimravids, barbourofelids, thylacosmilids). (b) Phylomorphospace depicted from the scores of the taxa taken from the first two axes of the analysis. Not all of the small conical‐toothed felids are shown, but they all cluster with the other small taxa with high scores on the second axis. (c) Representation of the variable loadings on both multivariate axes (see below for key). Modified from fig. 5 in Janis et al. (2020), by Science Graphic Design (sciencegraphicdesign.com): figure originally constructed by Borja Figueirido, used here with his permission. See Janis et al. (2020), for more details. Key to the morphological features: Cp, coronoid process; Ho, height of occiput; I, incisors; Io, infraorbital foramen; Js, jaw symphysis; Lc, lower canine; Lst, largest sectorial tooth; Mp, mastoid process; Npt, number of postcanine teeth; Pb, postorbital bar; So, shape of occiput; Ss, skull shape; Ucl, upper canine length. Silhouettes in (b) from phylopic. From left to right: *Panthera leo* by Lisa Nicvert (public domain), *Lynx rufus* by Margot Michaud (public domain), *Thylacosmilus atrox* by Ivan Ifrida (*attribution 4.0 International), *Homotherium venezuelensis* by Zimices (*attribution 3.0 Unported), *Barbourofelis fricki* by Zimices (*attribution 3.0 Unported). *See https://creativecommons.org/licenses/by‐nc/3.0/.

Simple linear measurements on sabertooth cranial proportions do not reveal differences between *Thylacosmilus* and placental sabertooths, despite the fact that morphological differences can be clearly observed. This is precisely because of the convergence due to the short rostrum and the wide gape. An alternative approach of performing a canonical correspondence analysis on a diversity of felids and felid‐like predators, including sabertooths (see Janis et al., 2020, for details and methodologies), does separate *Thylacosmilus* from the other sabertooths. As shown in Figure 5b, the first axis separates conical‐toothed felids (and the eupleurid *Cryptoprocta ferox*, the fossa) from all sabertooths. *Thylacosmilus* has the highest score along the first axis, separating it from all other sabertooths, including *Barbourofelis*, a taxon that others have considered to be the most similar to *Thylacosmilus* in form (e.g., Melchionna et al., 2021; in our analysis *Barbourofelis morrisi* clusters with the dirk‐toothed felids). The second axis separates the large pantherine cats (low scores) from the smaller felids and eupleurids, and the dirk‐toothed sabertooths (low scores) from the scimitar‐toothed ones (although *Thylacosmilus*, despite being dirk‐toothed, has a high score on this axis). *Thylacosmilus* is distinguished from other sabertooths primarily by the following features (see Figure 5c): the virtual lack of incisors, a low occiput, a very long and triangular‐shaped upper canine, and a very small lower canine.

A closer look at the craniodental morphology of *Thylacosmilus* shows that in various ways it does not appear to have had the capacity for either the proposed head strike or the canine shear bite, and there are many other aspects of its anatomy that are not indicative of proposed sabertooth‐like behavior. The following text and illustrations are taken from a collaborative analysis that investigated this mysterious metatherian predator in detail (Janis et al., 2020).

### 3.2 Capacity for dispatching prey in the proposed sabertoothed fashion

**FIGURE 6:**
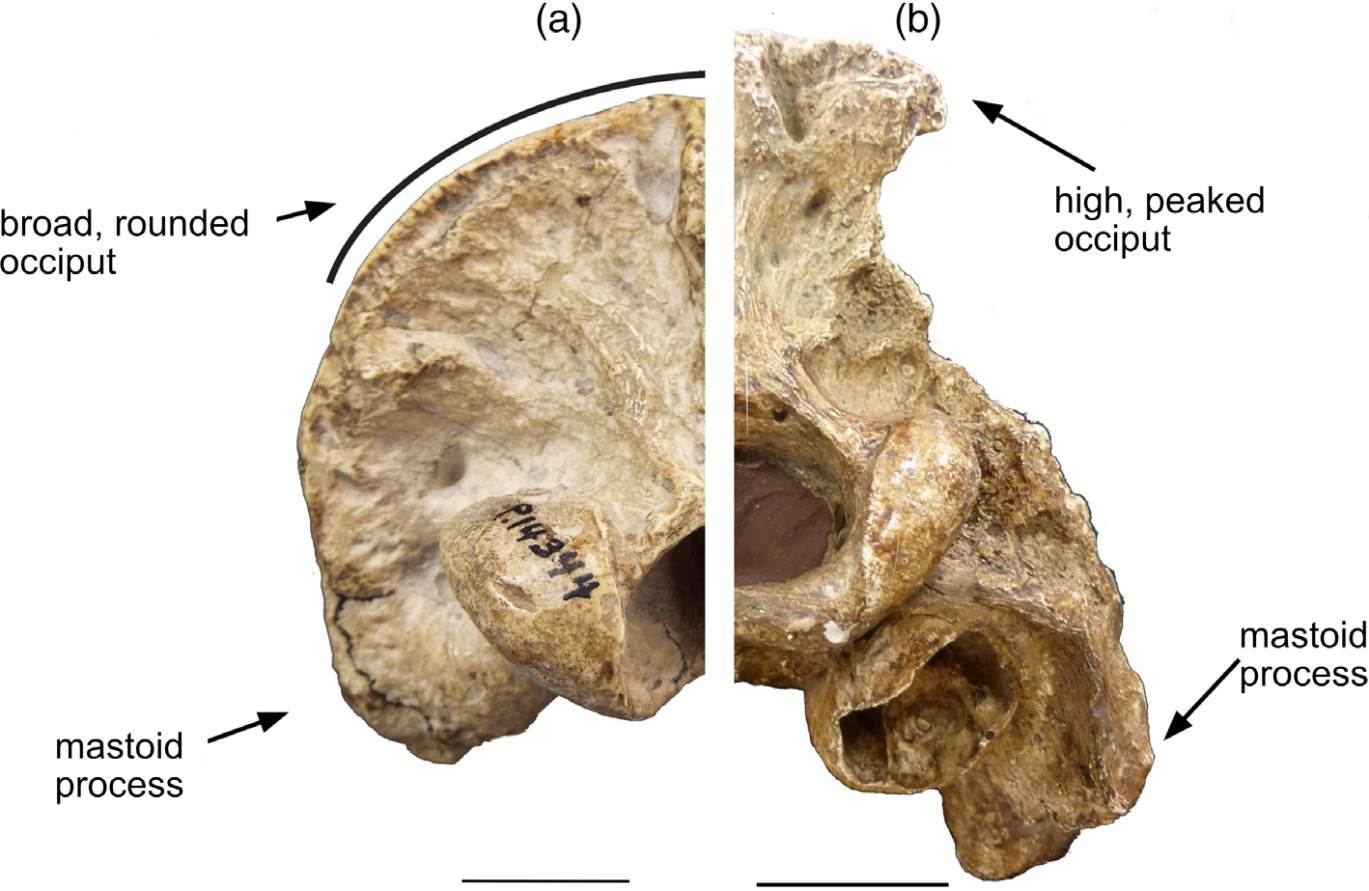
Comparison of occipital areas in (a) *Thylacosmilus atrox* (FMNH P14531‐holotype) and (b) *Megantereon cultridens* (AMNH 113842—felid) from Janis et al. (2020), fig. 2J (photos taken by Christine Janis). Figure modified by Science Graphic Design (sciencegraphicdesign.com).

Unlike sabertoothed felids, *Thylacosmilus* lacks both a high occipital area (nuchal crest) and a prominent mastoid process (see Figure 6), and thus would not have had the appropriate anatomy to produce a canine head strike. (Note that Turnbull, 1976, proposed that *Thylacosmilus* relied on another mode of head depression to the use of the sternomastoid, via the longis capitus inserting on the basisphenoid bosses, but this was comprehensively discussed and rejected in Janis et al., 2020). While the atlas wings of *Thylacosmilus* are relatively large, the small mastoid process could not have supported a sabertooth felid type of large obliquus capitus cranialis muscle, and so it would have been ill‐equipped to effect a canine shear bite.

Wroe et al. (2013) claim that *Thylacosmilus* had a more powerful head‐striking capacity for its size than *S. fatalis*, but their muscle reconstructions actually show much smaller head‐depressing muscles in *Thylacosmilus* (and, certainly, the areas of origin for such muscles are much smaller). Wroe and Sansalone (2023) resurrect this functional argument, but it is difficult to see how the occipital bony anatomy of *Thylacosmilus* could support this type of predatory behavior. The sternomastoid muscles would have been relatively small, and the head would have been at the wrong angle on the neck for the longis capitus (originating from the basisphenoid bosses, as proposed by Turnbull, 1976) to power a head strike (see also Emerson & Radinsky, 1980).

While the cervical anatomy of *Thylacosmilus* does indicate powerful neck muscles, they may not have been appropriate for the proposed sabertooth mode of killing prey. These muscles were positioned along the long axis of the neck, not suitable for powerful head elevation and depression but rather for resisting torsion and stabilizing the head on the neck (see discussion in Janis et al., 2020).

Other aspects of the anatomy of *Thylacosmilus* also do not reflect a felid‐like mode of predation. While the forelimbs were powerful, likely reflecting grappling ability (but not necessarily with live prey), the back was stiff, the limbs relatively short, the foot posture plantigrade, and the claws non‐retractile. Wroe and Sansalone (2023) note that a similar type of postcranial anatomy was present in the Eocene sabertoothed machaeroidine creodonts (Oxyaenidae, Machaeroidinae), such as *Machaeroides* and *Apataelurus*, and proposed that *Thylacosmilus* could have been a similar sort of sabertoothed predator. However, *Machaeroides eothan* had a body mass of around 12 kg (Egi, 2001)—i.e., about an order of magnitude smaller than *Thylacosmilus*—which does not make it a suitable predator for comparison.

### 3.3 The form of the canines

While *Thylacosmilus* did have enormous, blade‐like canines, there was some significant difference between these and those of sabertoothed felids.

**FIGURE 7:**
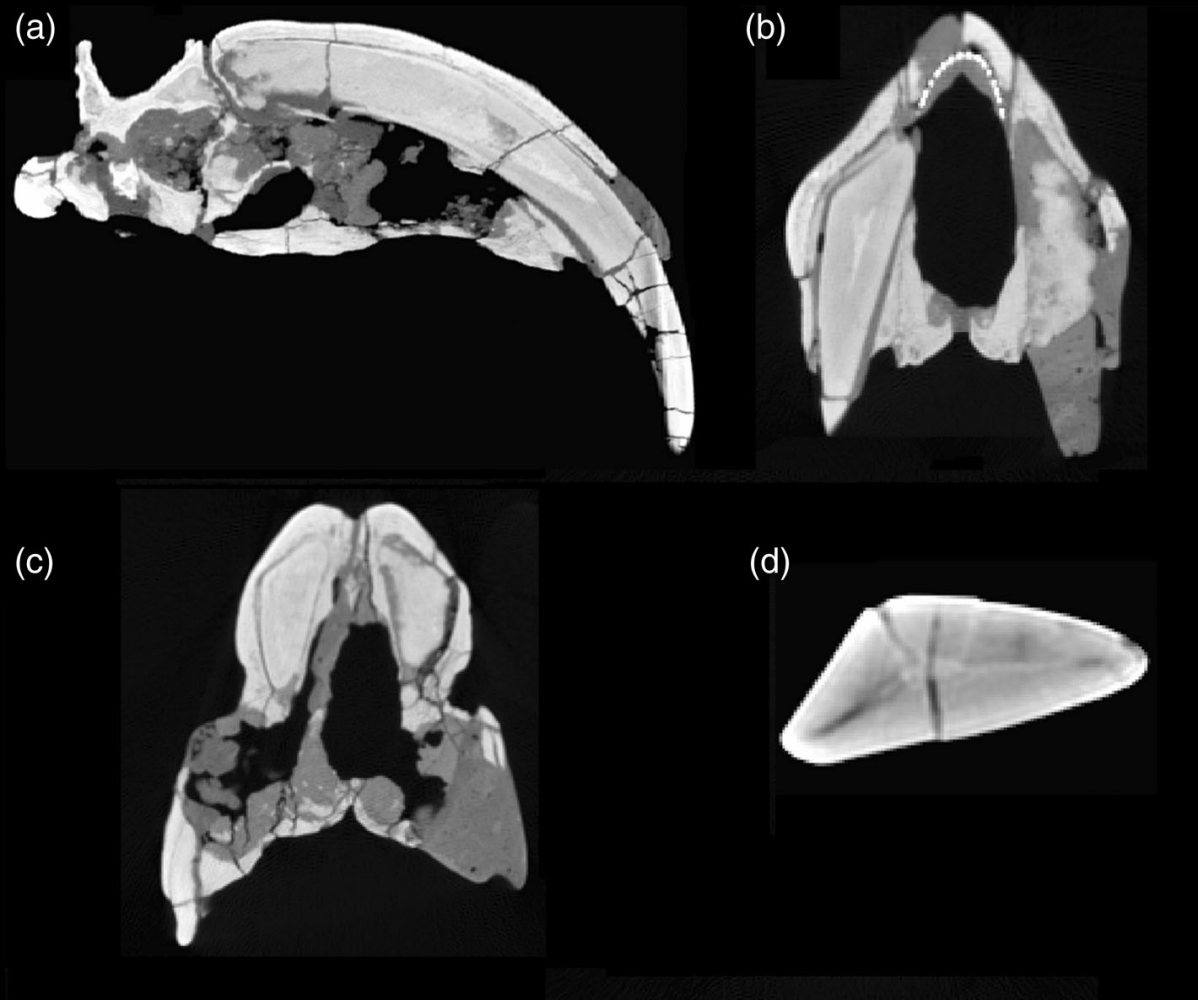
Sections through the cranium of *Thylacosmilus atrox* (CT scan of the holotype FMNH P‐14531). (a) Sagittal section, showing extreme posterior extension of the canines. (b) Cross‐section anterior to the orbits showing the slight divergence of the canines (the dotted lines represent a metal infilling, probably inserted during the restoration of the skull). (c) Cross‐section at around the level of the orbits showing the arched shape of the palate. (d) Cross‐section through the canine showing the triangular shape. From Janis et al. (2020), fig. 3 modified by Science Graphic Design (sciencegraphicdesign.com). [Correction added after first online publication on 10 May 2024. In the figure caption the FMNH value has been corrected.]

The ever‐growing nature of the canines can be seen in the fact that they are open rooted (Riggs, 1934: see also Figure 7a). Such canines have been thought by some to be indicative of a super‐sabertooth mode of life, but this more likely reflects the metatherian affinities of *Thylacosmilus*, with only a single lifetime set of teeth; ever‐growing canines are seen in smaller Miocene thylacosmilids (Suarez et al., 2023), in the borhyaenoid subfamily Proborhyaeninae (Bond & Pascual, 1983), and also in extant Australian marsupial carnivores (Jones, 2023). The canines, while blade‐like, are triangular (rather than oval) in cross‐section, with the apex of the triangle facing laterally, and the enamel is extremely thin (see Figure 7c). Enamel is found primarily on the lateral side of the canines and the canine tips do not show evidence of blunting, unlike the condition in other borhyaenoids. The general shape of the canines is more reminiscent of a giant claw than a blade (see later discussion of the performance of *Thylacosmilus* in “pull back” rather than in “stabbing”). However, the canines actually diverge slightly on their exit from the skull (there has been a good deal of debate about this, but Figure 7b of the type specimen shows that this was undoubtedly true). This divergence would make a simultaneous straight‐down strike with both canines impossible and make it likely that the canines were employed singly rather than together in any action.

### 3.4 Dental wear

The postcanine teeth of *Thylacosmilus* may have been sharp when they erupted (see plate I in Goin & Pascual, 1987), but they became blunt with wear (at least in the holotype, paratype, and one other specimen that I have seen [a commercial cast of an unnumbered skull]). This is not simply a matter of these being old individuals, as the carnassials of old felids show abrasion by shearing wear along the side, not tip wear on the occlusal surface.

**FIGURE 8:**
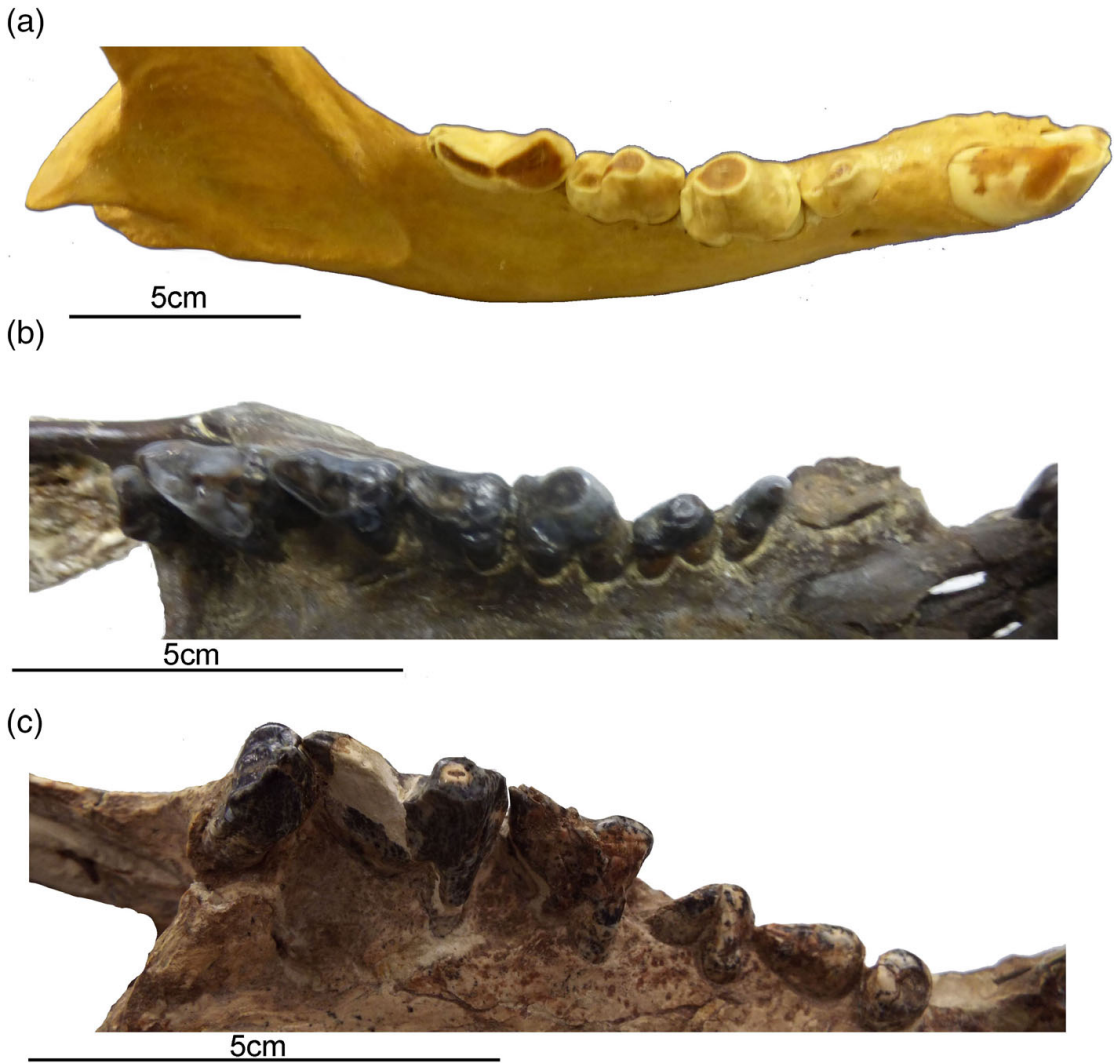
Comparison of gross dental wear in carnivorous mammals. (a) Right lower dentition of a spotted hyena (*Crocuta crocuta*, MCZ 50343), showing a clear distinction between shearing wear on the carnassial and tip‐crushing wear on the premolars. (b) Right upper dentition of a borhyaenoid sparassodont (Metatheria) (*Borhyaena tuberata*, YPM 15701), showing tip wear on the first molar (which resembles hyena premolars in form) and shearing wear on the posterior molars (especially evident on the third molar). (c) Right upper dentition of *Thylacosmilus atrox* (FMNH P14531, holotype) showing tip wear on the paracone of the third molar. Although there is some damage to the teeth, no evidence of carnivoran‐like shearing wear was observed on any of the teeth of the holotype or the paratype. From Janis et al. (2020), fig. 4. All photographs taken by CMJ.

Neither *Borhyaena* nor *Thylacosmilus* shows a reduced dentition with a single carnassial tooth like a placental carnivoran, but this reflects a general metatherian/eutherian difference. More relevant is the nature of the wear. The spotted hyena (*Crocuta crocuta*) shows clear crushing tip wear on the premolars (not seen in felids, which are not specialized for bone crushing) and shearing wear on the molars; *Borhyaena* shows a similar pattern of premolar/molar wear; in contrast, *Thylacosmilus* shows only tip‐crushing wear on both molars and premolars (see Figure 8).

The gross dental wear leads to the inference that *Thylacosmilus* was not eating fibrous food such as flesh (see below). While tip‐crushing wear is indicative of bone crushing in the hyena, and possibly also in *Borhyaena*, bones are unlikely to have been consumed by *Thylacosmilus* as the cranial anatomy shows evidence of weak jaw adductors, meaning that it could not have employed a powerful, hyena‐like bite with the posterior dentition.

**FIGURE 9:**
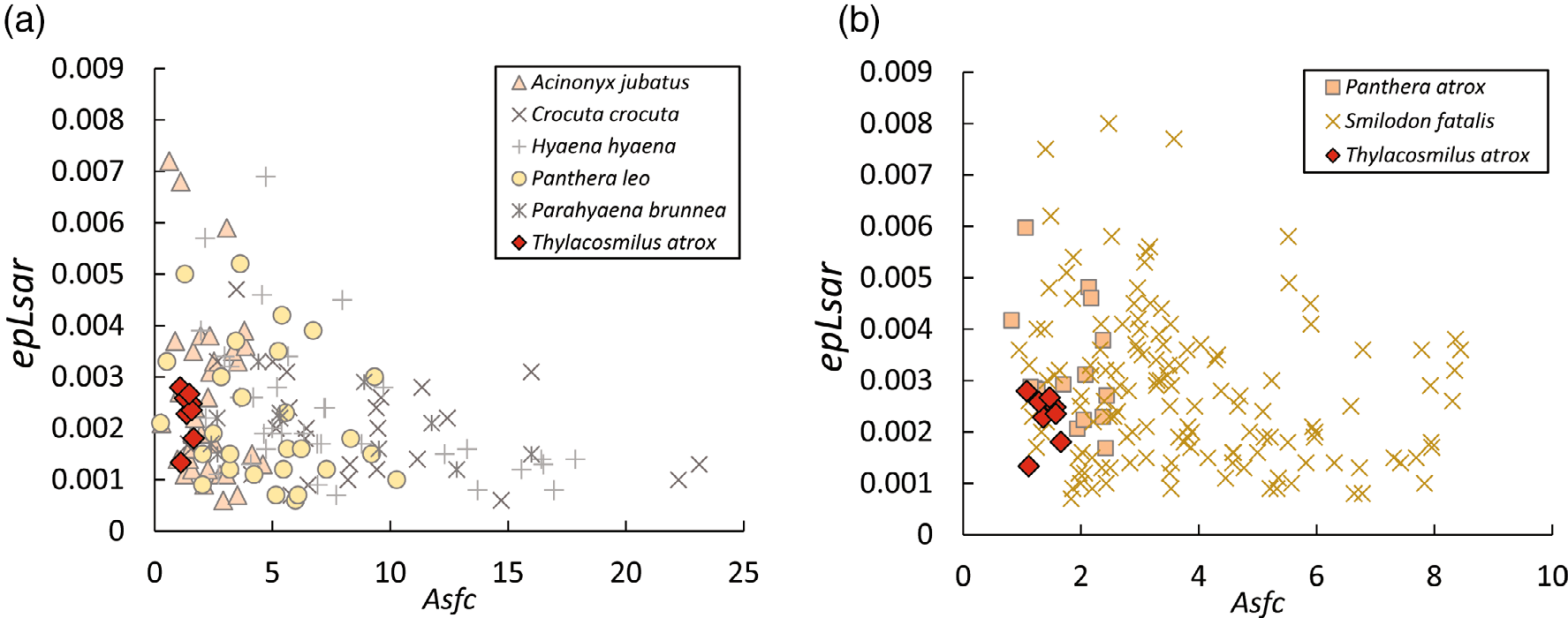
Dental microtexture analysis comparing *Thylacosmilus atrox* (red triangles) with a diversity of other carnivores. (a) Comparison with extant taxa. (b) Comparison with extinct taxa. Asfc = complexity, epLsar = anisotropy. From Janis et al. (2020), fig. 8, with permission from Larisa DeSantis.

Dental microtexture analysis (DMTA) backs up this conclusion (Figure 9, see Janis et al., 2020 for further details), showing that *Thylacosmilus* had low levels of both anisotropy and complexity, resembling the cheetah (*Acinonyx jubatus*) in a preference for soft, especially bone‐free food, and quite unlike both hyenas and extinct sabertooths such as *Smilodon*.

### 3.5 The nature of the incisors

**FIGURE 10:**
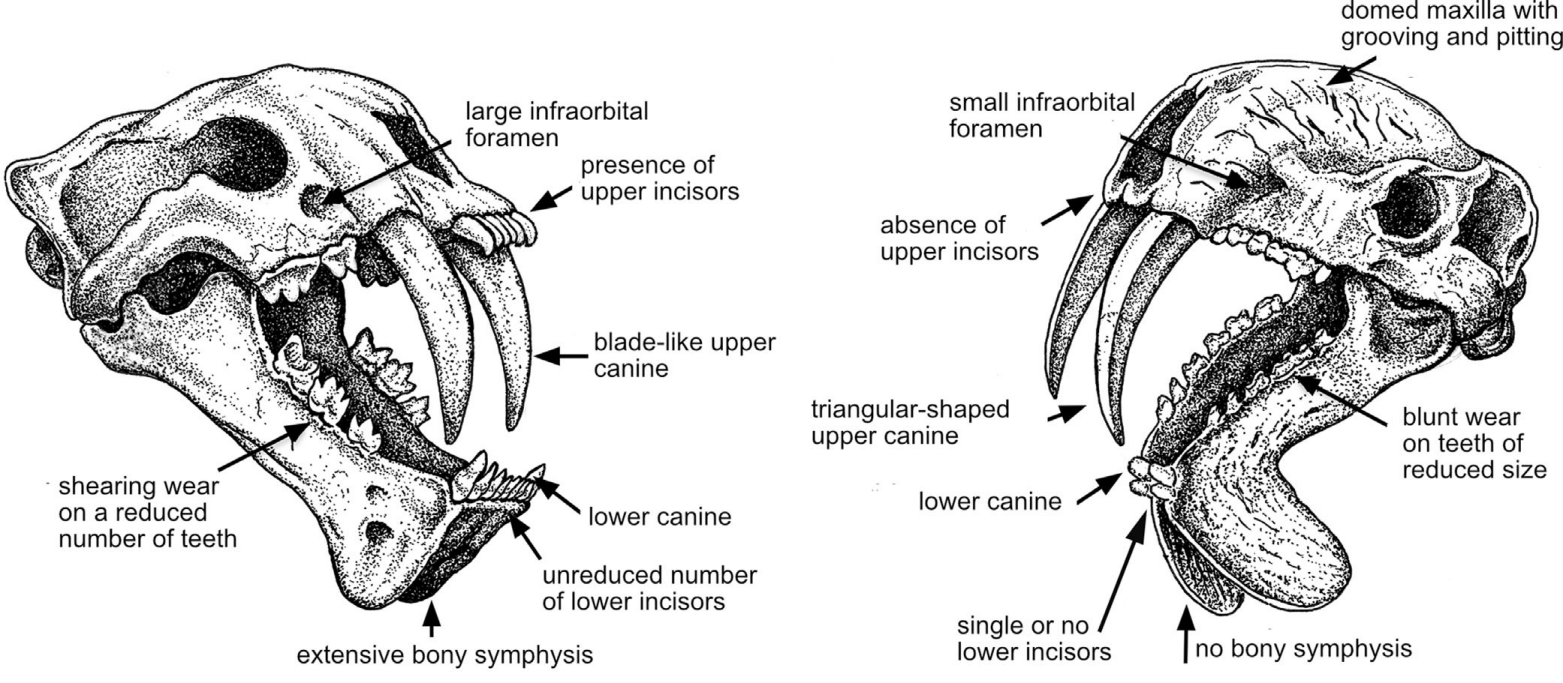
Comparison of the skulls of the “marsupial sabertooth” *Thylacosmilus atrox* (right) and the placental felid sabertooth *Megantereon cultridens* (left). Comparisons of sabertoothed skulls are usually portrayed in lateral view: this three‐quarter view better illustrates the differences between these two animals (the splaying of the symphysial flanges in *T. atrox* is exaggerated for effect to show the lack of bony connection: in life these flanges would have been appressed). Drawing by Michael Long. From Janis et al. (2020), fig. 1, modified by Science Graphic Design (sciencegraphicdesign.com).

Sabertoothed felids have a powerful incisor battery, with protruding incisors and a stout, but somewhat incisiform, lower canine. This incisor morphology is considered to reflect the ability to cut or scrape meat off the bone, as the large canines would impede the use of the carnassials for this purpose as seen in extant felids (Biknevicius et al., 1996). In contrast, *Thylacosmilus* appears to have lost its incisors almost entirely (see Figure 10). There is a small, peg‐like lower canine and, in some individuals, one or a pair of small lower incisors. Although the condition of the upper incisors cannot be known for certain, as no premaxillae are preserved, there would not be upper incisors without lowers to occlude with and the space between the canines is too narrow to accommodate an incisor array. The very fact that the premaxillae are missing may be an indication of the absence of upper incisors: among the skulls of extant mammals found in museums, ruminants, which lack upper incisors, are often missing the premaxillae whereas equids, that have strongly rooted upper incisors, are rarely missing this bone that anchors them (author's personal observation).

### 3.6 The size of the infraorbital foramen

Felids in general, and sabertoothed felids (and feliforms), have large infraorbital foramina. This foramen is for the passage of the maxillary nerve (cranial nerve V2) that carries sensory information back from the face. A large foramen, indicating a large nerve, indicates the importance of such sensory feedback in the precise placement of the canines, which would be particularly important if attacking live, struggling prey. In contrast, the infraorbital foramen of *Thylacosmilus* is small (see Figure 10), indicating a lesser amount of sensory feedback from the muzzle and hence a lesser ability for precise positioning of the canines.

### 3.7 The shape of the anterior mandible

While not all sabertooth felids have a prominent symphysial flange of the mandible (*Smilodon* being a prime example), all sabertooths have an extensive bony symphysis: this not only holds the lower incisor battery, but presumably bestowed increased strength during the proposed canine shear bite. While *Thylacosmilus* has a pronounced symphysial flange, there is no bony mandibular symphysis, unlike almost all other mammals (see Figure 10); other thylacosmilids were similar (Suarez et al., 2023). Some sort of tissue connection in life, perhaps cartilage, is indicated by the extensive pitting and bone sculpturing on the medial side of the flange.

### 3.8 Additional strange cranial features

The palate of *Thylacosmilus* is not flat, as in felids, but arched (see Figure 7c). The maxillae of *Thylacosmilus* are domed where they rise up over the top of the canines, accommodating the ever‐growing canines that have an origin quite a long way behind the level of the orbits (see Figure 7a), and there is a prominent patterning of grooves and pitting on these bones (see Figure 10). There may have been some soft tissue covering, possibly keratinous, over the top of the head in life. Gaillard et al. (2023) attribute this morphology, with the resulting telescoping of the neurocranium, as forcing the divergence of the orbits, unlike the more frontally‐positioned orbits typical of predators, and also seen in other sparassodonts.

Gaillard et al. (2023) also consider that this change in skull morphology explains the need for the postorbital bar in *Thylacosmilus*, to accommodate increased area for the origin of the temporalis muscle with the resultant reduced size of the temporal fossa. However, the skull has not been modified in this way in other extinct other cat‐like predators that have developed a postorbital bar: the barbourofelid feliform carnivoran *Barbourofelis* (although here the temporal fossa is shorter than in felid sabertooths) and the “marsupial lion” *T. carnifex*. The postorbital bar in *Thylacosmilus* might relate to strengthing the skull during “pull‐back” activity (see below).

### 3.9 Performance of the skull under stress

**FIGURE 11:**
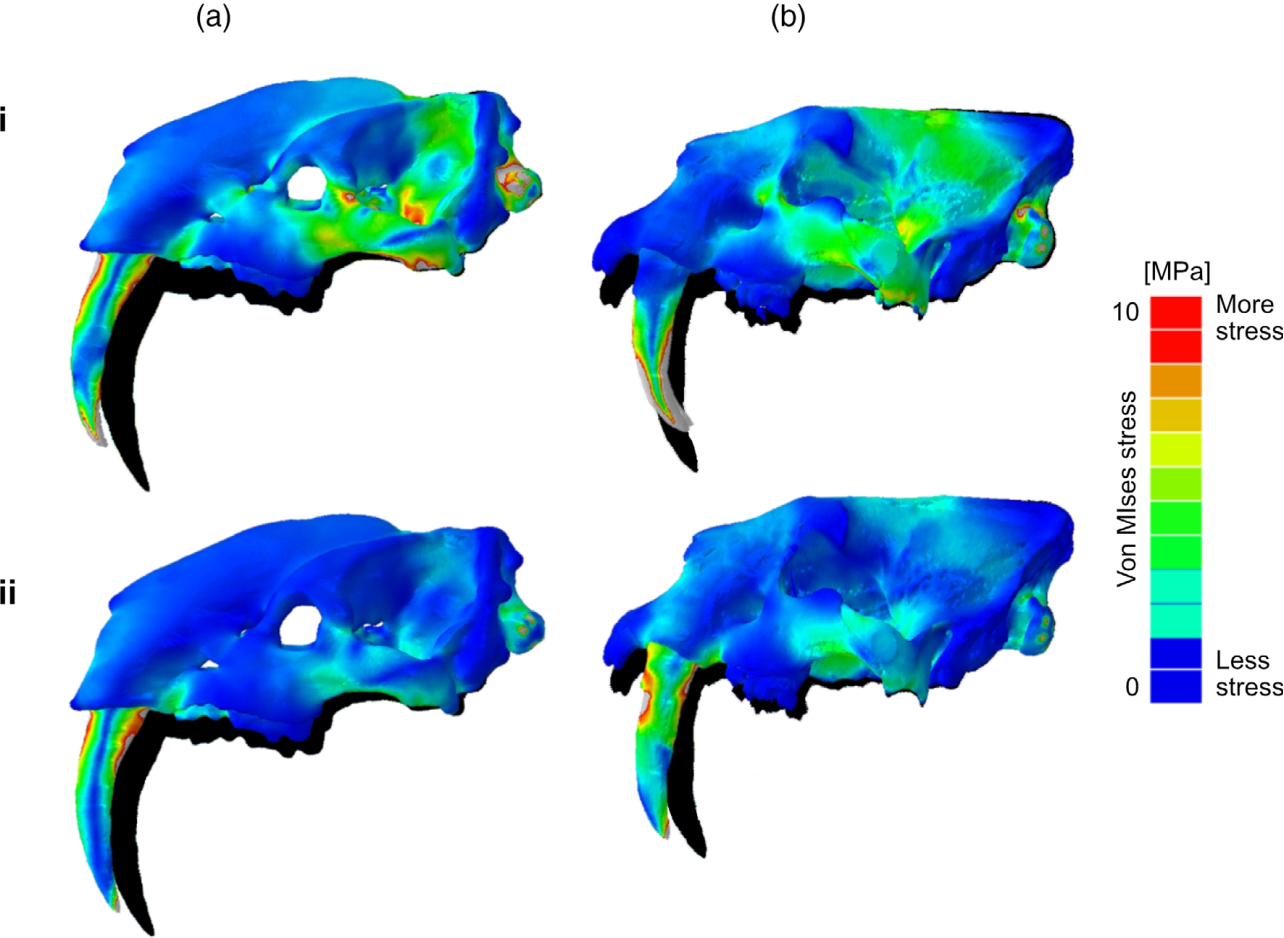
Finite element analysis stress patterns on skulls under different predation scenarios. (a) *Thylacosmilus atrox*, (b) *Smilodon fatalis*. (i) Stabbing, (ii) pull back. Modified from fig. 6 in Janis et al. (2020), by Stephan Lautenschlager, and used here with his permission.

Janis et al. (2020) presented a finite element analysis of the skull of *T. atrox* (FMNH P1531) in comparison with the sabertoothed felid *S. fatalis* (LACMRLP R37376): for details of the methodology see that paper. Three functionally different scenarios were tested, following Figueirido et al. (2018): stab, pull back, and shake. The first two simulations are of particular interest here (see Figure 11).

During the “stabbing” simulation, the performance of *Smilodon* is better than that of *Thylacosmilus*, while during the “pull back” one the reverse is true. The triangular shape of the canines of *Thylacosmilus* renders them better able to resist tensile and compressive stresses in all scenarios, especially along the lateral ridge. Experimental removal of the postorbital bar in *Thylacosmilus* results in greater deformation in both stabbing and pull‐back scenarios (see Janis et al., 2020).

### 3.10 Evolutionary history and probably paleobiology of *T. atrox*

Could *Thylacosmilus* be an end‐member specialist of a lineage that originally comprised more conventional sabertoothed predators? Other thylacosmilids are known from the Middle Miocene: *Anachlysictis gracilis* (see Goin, 1997), now known from a complete skull and some postcrania (Suarez et al., 2023), and *Patagosmilus goini* (see Forasiepi & Carlini, 2010), known from a rostrum. Both of these animals were considerably smaller than *Thylacosmilus*, being around 20 kg, and had upper canines that, while saber‐like in appearance, are considerably shorter (around half the length) than those of *Thylacosmilus*, and consequently, the symphysial flanges (known only in *Anachlysictis*) are smaller (Suarez et al., 2023). The skull of *Anachlysictis* is more like that of a typical carnivorous mammal, although not necessarily a specialized sabertoothed (see Suarez et al., 2023): the rostrum is longer, without the “telescoping” effect of the posterior extension of the maxilla over the orbits as in *Thylacosmilus*, and there is no postorbital bar; as in *Thylacosmilus*, the infraorbital foramen is small; upper incisors (3–4) are inferred to be present, but the condition is less clear for the lower incisors, although a relatively large lower canine is present; the molars are more “carnassialized” than in *Thylacosmilus*, with prominent protocones, and they appear to be relatively larger in comparison to the overall size of the animal; there are no prominent mastoid processes, and while there basisphenoid bosses are present, they are less prominent than in *Thylacosmilus*, so there is little indication of head strike specializations in this taxon. Thus, earlier thylacosmilids were certainly less derived in their cranial anatomy than *Thylacosmilus*, but they did not appear to be more specialized sabertoothed types of predators, although they may well have been predators of some sort.

In conclusion, what can be said about the probable lifestyle of *Thylacosmilus*? Despite the saber‐like teeth, it clearly did not have the predatory behavior of sabertoothed placental carnivores, as it lacks the anatomy for their proposed mode of killing prey. Its dentition resembles that of a carnivore in the unworn condition, but both the gross dental wear and dental microtexture wear indicate that it was eating soft food. While the DMTA shows it to be similar to a cheetah, the gross dental wear is counter‐indicative of eating fibrous flesh. Its non‐cursorial limb anatomy rules out sustained chases after prey; while the powerful forelimbs and neck musculature (Argot, 2004) show that it could have engaged in prey manipulation, the lack of retractile claws means that it would not have had the felid type of ability to restrain struggling prey.

My preferred scenario is that it was a type of scavenger specializing in the soft parts of prey (i.e., internal organs). Although no carnivorous mammal today is entirely dependent on scavenging, it must be remembered that marsupials (and so, presumably, all metatherians) have a lower metabolic rate than placentals (McNab, 1986); perhaps the daily metabolic needs of *Thylacosmilus* were lower than that of a placental carnivore of equivalent size. A preference for internal organs could account for many of the anatomical features of *Thylacosmilus*: the dental wear indicating soft food, the pull‐back capacity of the skull and claw‐like canines that would enable the opening of carcasses, and the powerful forelimbs that would aid in the manipulation of large carcasses. *Thylacosmilus* lived in sympatry with a diversity of large carnivorous birds (Phorisrachiformes), and perhaps it depended on these birds to make the initial killing.

*Thylacosmilus* would have been extremely unlikely to have deployed these canines on the belly of a living prey item, even if the somewhat divergent canines would have allowed for a lateral strike at the flank. While this is indeed the mode of attack of the Komodo Dragon (*Varanus komodoensis*), which ambushes and slashes its prey and then waits for it to succumb, this reptile has venom in its bite which means that its victim rapidly succumbs to shock, and does not wander off out of distance (Fry et al., 2009); this does not appear to have been a likely option for *Thylacosmilus*. Moreover, the very long and thin canines of *Thylacosmilus* would be at high risk of breakage with such behavior (see Van Valkenburgh & Ruff, 1987), especially as it lacked felid‐like retractile claws, and so would not have been as adept at immobilizing is prey.

Wroe and Sansalone (2023) note that it would be difficult for such a scavenger to persist, as the initial predators (i.e., phorisrachiform birds) would surely open up the carcass of the prey, thus obviating the need for *Thylacosmilus* to use its canines for this purpose. These birds would also prefer the internal organs, thus leaving little for a scavenger to eat if it did not utilize the entire carcass, including the bones (and the dental wear of *Thylacosmilus* rules out bone crushing). While these authors have a good point, in truth the ecology of the Pliocene of Argentina is both unknown and unknowable: no ecomorphological analogs to either the large birds or the metatherians of that time exist today. My hypothesis about the possible behavior of *Thylacosmilus* is based entirely on its anatomy, which leads me to conclude that it was a carnivore that, despite its fearsome canines, did not use those teeth to kill other animals.

Additional strange aspects of the cranial anatomy of *Thylacosmilus* may provide further clues about its behavior, although here we are wandering heavily into the land of speculation. The lack of incisors and the arched palate resemble the anatomy seen today in mammals that have a large fleshy tongue that they use for food manipulation (Davit‐Béal et al., 2009; Gordon, 1984). Perhaps *Thylacosmilus* employed such a tongue to “slurp up” the innards of prey, although such a scenario would be difficult to formulate as a testable hypothesis.

## 4 THE “MARSUPIAL LION” *THYLACOLEO CARNIFEX*

**FIGURE 12:**
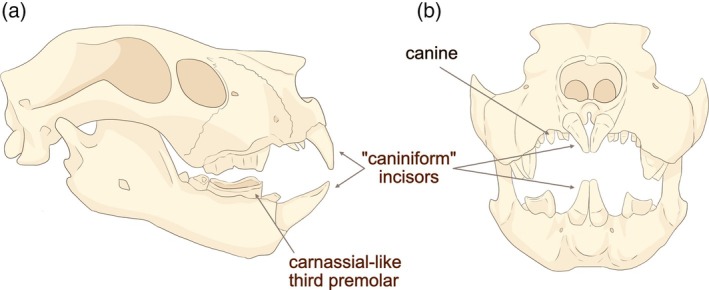
Skull of *Thylacoleo carnifex*. (a) Lateral view, (b) anterior view. (a) Modified from Woodward (1896), fig. 102, with details of the third premolar from Owen (1887), fig. 1. (b) Modified from Encyclopedia Britannica (1911). Figures redrawn by Science Graphic Design (sciencegraphicdesign.com).

*Thylacoleo carnifex* was the Pleistocene end‐member of the family Thylacosmilidae (Diprotodontia), first known from the Early Miocene. The Miocene genera were considerably smaller in size (590 g–38 kg; Gillespie, 2023; Gillespie et al., 2016) than the Plio‐Pleistocene species of *Thylacoleo. Thylacoleo* was clearly not a sabertooth as it lacked saber‐like canines: the enlarged first incisors (the others are highly reduced or absent, Wells et al., 1982) are often said to be “caniniform,” but see discussion below as to their probable function—they did not function like felid canines. A tiny upper canine is present (see Wells et al., 1982); these canines were larger (but never very large) in some earlier members of the family (Gillespie, 2007). But *Thylacoleo* was clearly an impressive predator, with an extremely powerful bite (Wroe, 2008; Wroe et al., 2008; Wroe & Sansalone, 2023), and sports a huge carnassial‐like tooth in each jaw half (= the third, or last, premolar, the other premolars and the molars are greatly reduced) that dominates the short jaws (see Figure 12). Despite some earlier speculations that these carnassial‐like teeth were used to cut up large fruits (see discussion in Anderson, 1929), a proposal similar to that of the creationists for the use of *Tyrannosaurus rex* teeth before the Fall of Man, when all animals were supposedly herbivorous, these teeth show clear evidence of shearing wear indicative of processing flesh (Wells et al., 1982; see also Figure 12a), in contrast to the blunted postcanine teeth of *Thylacosmilus*. The gross dental wear on the carnassials also shows a pattern of relatively broadly placed striae, similar to known carnivorous placentals (Wells et al., 1982).

### 4.1 Craniodental indicators of predatory behavior in *Thylacoleo*

**FIGURE 13:**
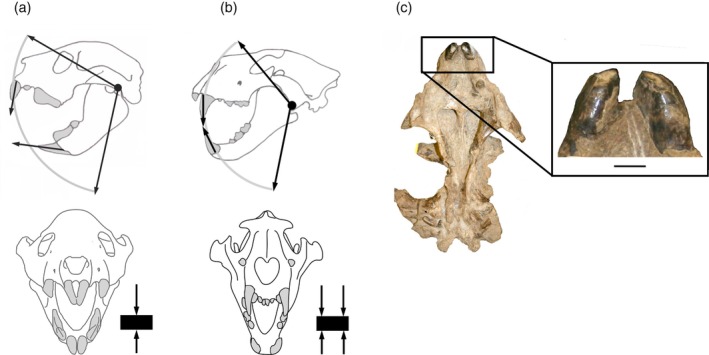
Lateral and anterior views of the skulls showing occlusion of (a) *Thylacoleo carnifex*, (b) Placental lion (*Panthera leo*). (c) View of upper incisors of *T. carnifex* (AMNH 19251) showing blunt wear. Modified from Figueirido et al. (2016), fig. 10, by Science Graphic Design (sciencegraphicdesign.com), with permission from Borja Figueirido and Cambridge University Press.

There is little doubt today that *Thylacoleo* was a hypercarnivore, with a powerfully built and muscular body (Wells & Camens, 2018). But how did it dispatch its prey? While its large incisors (resembling those of many other diprotondontid marsupials) have been termed “caniniform,” it appears that they could not have been used to kill prey in the manner of placental carnivore canines. The upper incisors at least wear to be blunt (unlike carnivoran canines) and the incisors meet at the tip, rather than sliding past the lowers to form a shearing surface as with carnivoran canines (see Figure 13).

Sir Richard Owen (1871) noted the similarity of the skull of *Thylacoleo* to the (much smaller) skull of the Aye‐Aye (*Daubentonia madagascariensis*), an extant lemur that uses its similarly‐shaped incisors to grip bark. The incisors of *Thylacoleo* may have been deployed to grip the skin of the prey, either to subdue or to manipulate it, but it is clear that they cannot have been used for piercing the skin, and so not used for killing the prey (see discussion in Figueirido et al., 2016). Wells et al. (1982) note that the lower incisors maintain a sharp occlusal surface with wear, and might have had a piercing or stabbing function with the upper incisors acting as an anvil.

How, then, might *Thylacoleo* have killed its prey? The huge carnassial‐like teeth could not have been used in a cat‐like killing bite, but might have been used to open up wounds causing blood loss leading to death (Wroe, 2008), or the caniniform incisors might have been used to crush the skull or perform a suffocating throat bite (Wells et al., 1982; Wroe & Sansalone, 2023). Wroe and Sansalone (2023) also claim that the powerful bite of *Thylacoleo* could have affected a bite through the throat, the large incisors serving as guides for the slicing premolars behind them. Cut marks found on ribs and limb bones, previously ascribed to human activity, have now been identified as being made by *Thylacoleo* carnassia‐like premolars (Horton & Wright, 1981; Wells et al., 2006). It is possible that the marks on the limb bones might represent predatory attacks, rather than consumption of the prey.

### 4.2 Postcranial indicators of predatory behavior in *Thylacoleo*

**FIGURE 14:**
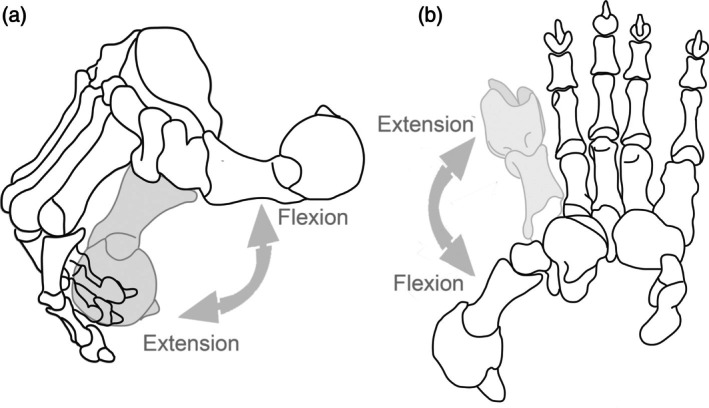
*Thylacoleo carnifex* manus, showing hypothesized movements of the pollux. Right manus with digits II to V flexed and digit I showing flexion‐extension in (a) medial view, (b) dorsal view. Modified by Science Graphic Design (sciencegraphicdesign.com) from Figueirido et al. (2016), fig. 2 (originally modified from a figure in Wells & Nichol, 1977), with permission from Borja Figueirido and Cambridge University Press.

**FIGURE 15:**
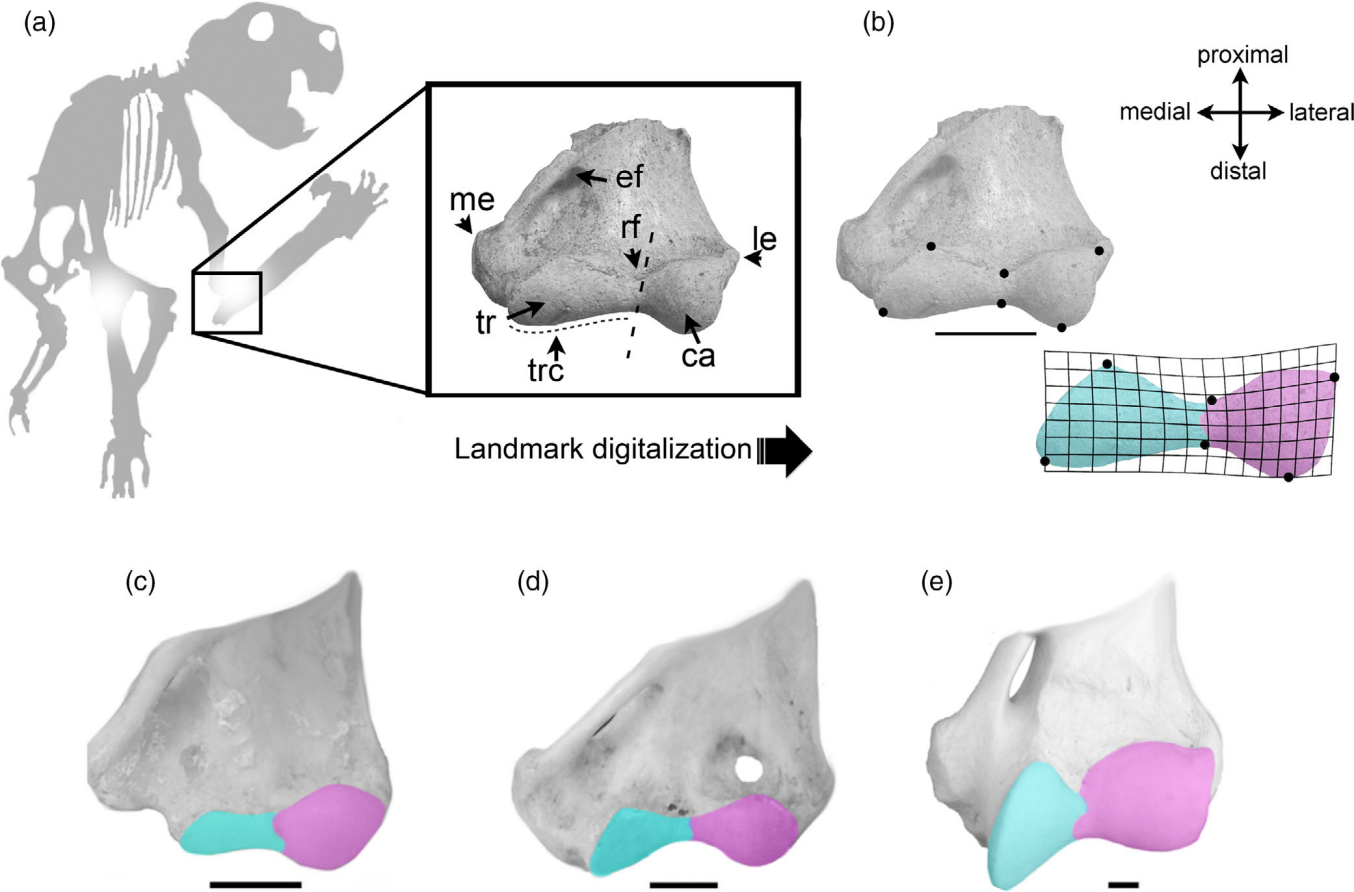
Distal humerus articular morphology of *Thylacoleo carnifex* (SAM‐P12384b) and comparison with some other mammals. (a) Orientation of *Thylacoleo* elbow joint, and details of anatomy. Ca, capitulum; Trc, trochlea. (b) Details of landmarks placed and schematic picture of morphology: long, somewhat expandeded trochela, somewhat rounded capitulum. (c) Distal humerus of an arboreal marsupial with a highly mobile elbow joint, the koala (*Phascolarctos cinereus*). Note the short, slender trochlea and the highly rounded capitulum. (d) Distal humerus of the common wombat (*Vombatus ursinus*), with an elbow somewhat similar to *Thylacoleo*. (e) Distal humerus of a terrestrial carnivoran with a less mobile elbow joint, the tiger (*Panthera tigris*). Note the long, deflected trochlea and the square capitulum. From Figueirido et al. (2016), fig. 1 (in part) and fig. 2, modified by Science Graphic Design (sciencegraphicdesign.com), with permission from Borja Figueirido and Cambridge University Press.

Another possibility is that *Thylacoleo* used the hypertrophied claw (dew claw) on its semi‐opposable thumb (see Figure 14) to kill its prey (Figueirido et al., 2016). *Thylacoleo* also had an unusual type of elbow morphology for a carnivore: geometric morphometric analysis of the articulation of the distal humerus shows that it had both the capacity for terrestrial support and for a high degree of supination, unlike any extant placental carnivore that has specialized its forelimbs to a greater extent for terrestrial support (Figure 15: Figueirido et al., 2016, and see that paper for discussion, methodology, and further details). This elbow joint may in part reflect the arboreal behavior of earlier thylacoleonids (see Gillespie, 2007), but it still bestowed a unique capacity for forelimb use in this animal in comparison to placental carnivores. The supinatory properties of the elbow joint of *Thylacoleo* could certainly have aided the function of a large claw on the pollux in dispatching prey, while still supporting a primarily terrestrial predator.

Wroe and Sansalone (2023) are skeptical of this hypothesis, noting that the size of the claw on the pollex of *Thylacoleo* is not that absolutely large, compared with the dew claws of large felids; the claw appears to be so large because the other manual claws are relatively small, but the absolute length (estimated as a maximum 28 mm) would be insufficient to dispatch large prey. Wroe and Sansalone (2023) also note that some sabertoothed felids, such as *S. fatalis* and *Homotherium latidens*, also had hypertrophied dew claws, suggesting that all of these carnivores (and also large living felids) used such claws to gain purchase on struggling prey, or perhaps also for climbing. But these felids lacked the opposable pollux and the elbow capable of the degree of supination seen in *Thylacosmilus*, so the function of the claw in the marsupial predator was likely to have been different from in the placentals. It is certainly possible that *Thylacoleo* could have used its teeth in some fashion to kill its prey, but its forelimb also appears to be uniquely specialized in a manner that may have aided prey killing in a manner unlike that seen in placental cat‐like carnivores.

While the mode of prey killing of *Thylacoleo* is unknown, and indeed unknowable, it does appear to have been a powerful predator, perhaps capable of bringing down prey larger than itself as proposed for sabertooth predators.

## 5 DISCUSSION

*Thylacosmilus* and *Thylacoleo* are both charismatic and enigmatic animals that have captured the public imagination, as “marsupial” versions of well‐known placental carnivores, either extant (lions) or recently extinct (sabertoothed “tigers”). But a more detailed consideration of their anatomy shows that they cannot be easily analogized with such placental mammals.

*Thylacosmilus* did indeed have saber‐like canines and has been portrayed as being even a more extreme type of sabertoothed predator than placental sabertooths (e.g., Wroe et al., 2013; Wroe & Sansalone, 2023). But other aspects of the anatomy of *Thylacosmilus*, both craniodental and postcranial, are not at all like those of sabertoothed felids, and it lacks many of the critical features that would enable it to kill its prey in the manner proposed for placental sabertooths. Even if more recent considerations of this animal (Suarez et al., 2023; Wroe & Sansalone, 2023) disagree with the premise that it was a scavenger rather than an active predator, as proposed by Janis et al. (2020), these authors do admit that it was an odd beast, and rather different to a placental sabertooth.

A couple of critical features, to my mind, that are often overlooked are the small infraorbital foramen and the virtual lack of incisors, in which way it is profoundly different from placental sabertooths. How was *Thylacosmilus* able to have the sensory feedback for precise placement of the canines (essential if they were used for killing prey) and how did it get meat off the bone? There are only a handful of skulls known for this animal, but I have never heard of a report of a broken canine, although this is not that an uncommon event for *S. fatalis* (Van Valkenburgh & Hertel, 1993, although this is from sampling very large numbers of individuals at the La Brea tarpits).

To my mind, *Thylacosmilus* does not qualify as an ecological analog of placental sabertooths. While there are admittedly a number of problems with the notion that it was a scavenger rather than an active predator, it could not have had the same sort of predatory behavior as the placental sabertooths—its anatomy is just too different.

In contrast, although *Thylacoleo* virtually lacked canines entirely, and so cannot technically be considered to be any sort of sabertooth predator, in many respects its anatomy indicates a type of predatory lifestyle similar to that proposed for sabertoothed placentals: that of a very powerful predator that was easily able to tackle large prey while hunting alone. While there is still considerable debate about how *Thylacoleo* dispatched its prey, I would give my vote to this animal being the “true sabertooth” out of the two metatherians, in terms of ecomorphological similarities (albeit with different anatomy) and role in the ecosystem. But neither metatherian can really be simply assigned as the “marsupial” version of a placental counterpart. The unique attributes of extinct animals should be appreciated, and attempts to shoehorn them into more familiar ecomorphological roles should be cautioned.

## AUTHOR CONTRIBUTIONS

**Christine M. Janis:** Conceptualization; investigation; writing—original draft; visualization (preparation for Science Graphic Design); writing—review and editing; supervision.

## CONFLICT OF INTEREST STATEMENT

The author declares no conflict of interest.

## Data Availability

All data are included in the manuscript or can be found in the papers by Figueirido et al. (2016) and Janis et al. (2020) (see full citations below).
